# Exercise Training and Cardiovascular Risk Factors in Males with Overweight or Obesity: A Systematic Review of Randomized Controlled Trials

**DOI:** 10.3390/medicina61020255

**Published:** 2025-02-01

**Authors:** Aynaz Pourmotahari, Shahnaz Shahrbanian, Rashmi Supriya, Ayoub Saeidi

**Affiliations:** 1 Department of Sport Sciences, Faculty of Humanities, Tarbiat Modares University, Tehran P.O. Box 14115-111, Iran; ainaz_motahari@yahoo.com; 2 Department of Sport, Physical Education and Health, Academy of Wellness and Human Development, Faculty of Arts and Social Sciences, Hong Kong Baptist University, Kowloon Tong, Hong Kong 999077, China; 3 Department of Physical Education and Sport Sciences, Faculty of Humanities and Social Sciences, University of Kurdistan, Sanandaj P.O. Box 66177-15175, Iran; a.saeidi@uok.ac.ir

**Keywords:** lipid profile, blood pressure, insulin, glucose, combined training, exercise, high-intensity interval

## Abstract

*Background and Objectives:* Obesity is a significant risk factor for cardiovascular disease. Physical exercise has been established as an effective intervention for reducing cardiovascular mortality. This systematic review aimed to investigate the effects of various exercise modalities on cardiovascular risk factors in men with obesity. *Materials and Methods:* This review included randomized controlled trials (RCTs) published between 2005 and November 2023. Studies were eligible if they assessed the impact of exercise interventions on blood pressure (BP), lipid profiles, and glucose/insulin regulation in males aged 18 years or older with a body mass index (BMI) of 25 kg/m^2^ or greater. The quality of included studies was evaluated using the Physiotherapy Evidence Database (PEDro) scale. *Results:* A total of 25 RCTs were included in the analysis, with PEDro scores ranging from 3 to 7. High-intensity interval training (HIIT) was associated with significant reductions in BP, with an average decrease of 12.5 mmHg. However, the magnitude of BP reduction varied across studies, likely due to differences in training protocols and study quality. Resistance training (RT) was associated with modest improvements in glycemic control, with some studies reporting a reduction in fasting blood glucose levels by 5–10%. Combined exercise (CT) programs, which included both aerobic and resistance training, as well as aerobic exercise alone, were shown to improve lipid profiles, with some studies showing reductions in total cholesterol levels ranging from 10–15%. However, the findings were inconsistent, highlighting the need for further research to better understand the potential benefits and optimal exercise regimens. *Conclusions:* This systematic review supports the potential of exercise training in reducing cardiovascular risk factors in men with obesity, though the effectiveness varies depends on the type of exercise. Combined exercise programs have shown promise for lipid profiles, but further research is needed to confirm these effects. HIIT has been linked to BP reductions in some studies, although the results are inconsistent and depend on protocols and study quality. The limitations in study quality may impact the reliability of these findings.

## 1. Introduction

Obesity, defined as a body mass index (BMI) of 30 kg/m^2^ or greater, is a chronic condition with profound health and economic consequences. Its global prevalence has risen sharply in recent decades. According to the World Health Organization (WHO), 43% of adults were overweight in 2022, with 16% classified as obese. Obesity rates are particularly high in North America, Europe, and the Western Pacific, but low- and middle-income countries are also experiencing significant increases [1,2]. Projections suggest that by 2030, 20% of women and 15% of men globally will be affected by obesity [3], contributing to as much as 2.8% of national healthcare costs [4].

Obesity is a critical risk factor for the development of various chronic diseases, including cardiovascular disease (CVD), type 2 diabetes, and certain types of cancer [5]. Given its strong association with hypertension and dyslipidemia, obesity is recognized as one of the top 10 CVD risk factors by the American Society for Preventive Cardiology (ASPC) [6].

The health risks associated with obesity increase proportionally with higher BMI [7]. These risks are closely linked to metabolic disturbances, such as altered glucose and fat metabolism [8] and elevated BP [9]. While the association between weight gain and hypertension is well-established, the precise underlying mechanisms, including insulin resistance and hyperinsulinemia, remain subjects of ongoing investigation [7].

Physical inactivity exacerbates obesity-related health risks and is a leading cause of preventable mortality worldwide [10]. Exercise, particularly aerobic exercise and RT, is an essential component in the management of overweight and obesity [11]. Aerobic exercise can help reduce visceral fat, improve lean body mass, lower BP, enhance lipid profiles, and decrease triglyceride levels, all of which may contribute to better cardiovascular health [12]. The World Health Organization (WHO) underscores aerobic exercise as a beneficial approach to supporting cardiovascular health. Activities such as walking, cycling, and swimming are believed to contribute to improvements in blood pressure, triglyceride levels, and HDL cholesterol. Evidence indicates that regular aerobic exercise might help lower triglycerides by 10–20%, which could have a positive impact on cardiovascular risk in individuals with obesity [13].

Resistance training (RT), while not as immediately impactful as aerobic exercise on certain cardiovascular markers, has been associated with potential benefits such as maintaining muscle mass, promoting insulin sensitivity, and influencing metabolic rate. These factors may contribute to the management of obesity over time [14].

High-intensity interval training (HIIT), which involves short bursts of intense activity followed by recovery periods, has been suggested to improve vascular function and lead to modest reductions in blood pressure, potentially offering benefits beyond those of traditional moderate-intensity exercise. Some studies indicate that HIIT might help reduce visceral fat and modestly enhance insulin sensitivity, which could contribute to better regulation of blood glucose levels in individuals with obesity [15]. However, due to its higher intensity, HIIT may not be ideal for everyone, particularly individuals with specific cardiovascular conditions, and its demanding nature could pose challenges for adherence and long-term sustainability [16].

Combining aerobic exercise and RT is considered a potentially effective strategy for supporting metabolic health and addressing multiple risk factors. Some studies suggest that this combined approach might contribute to improvements in body composition, insulin sensitivity, and lipid profiles compared to focusing on a single type of exercise [17].

Despite these findings, much of the existing literature has concentrated on isolated exercise modalities, with relatively limited exploration of how combined exercise strategies influence cardiovascular risk factors in individuals with overweight or obesity. This gap highlights an opportunity for a more comprehensive understanding of how diverse exercise approaches can synergistically address metabolic health. While previous reviews have predominantly focused on individual exercise types, this review uniquely emphasizes the comparative effects of HIIT, MICT, CT, and resistance training RT on cardiovascular health indicators, such as lipid profiles, BP, glucose regulation, and insulin sensitivity. Given the widespread prevalence of obesity and its well-documented association with cardiovascular risk, understanding the relative and combined impacts of different exercise modalities is an important area of study.

This systematic review aims to evaluate the effectiveness of various exercise interventions in improving cardiovascular risk factors—specifically lipid profiles, blood pressure, BP levels, and insulin sensitivity—in men with overweight or obesity. By synthesizing the available evidence, this study aims to offer informed recommendations for exercise prescriptions tailored to this population.

## 2. Methods

This systematic review was conducted following the guidelines provided by the Preferred Reporting Items for Systematic Reviews and Meta-Analyses (PRISMA) statement [18].

### 2.1. Data Sources and Search Strategy

Two researchers independently conducted a comprehensive search for studies published between 2005 and November 2023. The following electronic databases were searched systematically: PubMed, Web of Science, PEDro, ScienceDirect, and Google Scholar. The search strategy used a combination of relevant terms related to exercise, cardiovascular risk factors, and obesity. The key search terms included the following:

Exercise-related terms: “Exercise training”, “exercise”, “training”, “resistance training”, “strength training”, “aerobic exercise”, “aerobic training”, “high-intensity interval training”, “moderate-intensity continuous training”, “HIIT”, “HIT”, “MICT”, “physical activity”.

Cardiovascular risk factors: “lipid profile”, “HDL”, “LDL”, “VLDL”, “cholesterol”, “triglycerides”, “TG”, “high-density lipoprotein”, “low-density lipoprotein”, “very-low-density lipoprotein cholesterol”, “insulin”, “insulin resistance”, “insulin sensitivity”, “glucose”, “glucose tolerance”, “blood pressure”, “systolic BP”, “diastolic BP”.

Obesity-related terms: “Obese males”, “obese male”, “obesity”, “overweight males”.

The search terms were combined using Boolean operators, such as AND to link different concepts (e.g., “exercise AND cardiovascular risk factors AND obesity”), and OR to include synonyms for key terms. Only studies published in English were considered.

### 2.2. Eligibility Criteria

To be eligible for inclusion, studies had to be randomized controlled trials (RCTs) that compared the effects of any type of exercise training (aerobic, RT, HIIT, MICT, or a combination of exercises) against a no-exercise control group or another exercise modality. Studies must have provided detailed information about the type, intensity, frequency, and duration of the exercise intervention.

The following inclusion criteria were applied:

Participants: Males aged 18 years or older with a body mass index (BMI) ≥ 25, classified as overweight or obese.

Interventions: Studies must involve structured exercise interventions with specified parameters (type, intensity, duration, and frequency).

Control Group: Studies must include a comparison group that did not engage in any exercise or involved a different exercise modality.

Outcomes: Studies must report on cardiovascular risk factors such as lipid profiles, blood pressure (BP), blood glucose, and insulin sensitivity.

### 2.3. Exclusion Criteria Included

Single-session interventions: Studies that involved one-off interventions were excluded. We did not aim to investigate the acute effects of a single exercise session, as single-session interventions often lack the necessary duration and consistency to produce meaningful, lasting changes in cardiovascular risk factors. In fact, our goal was to identify exercise interventions that lead to long-term improvements in cardiovascular risk factors. Numerous studies have highlighted the importance of the frequency, intensity, and duration of exercise in determining its effectiveness.

Participants with existing cardiovascular or other chronic diseases: Studies that involved participants with diagnosed cardiovascular diseases, diabetes, or other major chronic conditions were excluded unless the study provided separate data for healthy male participants.

Mixed-gender studies: Studies that reported combined results for both male and female participants were included only if data specific to males were available.

Non-RCT studies: Only randomized controlled trials were included in the review.

Duplicates and studies not meeting the eligibility criteria were excluded during the initial screening process.

### 2.4. Data Extraction and Verification

Data from eligible studies were independently extracted by two reviewers (Author 1 and Author 2) using a standardized form. The following data were extracted: study characteristics (authors, year of publication, sample size, and demographic information), details of the exercise intervention (type, frequency, intensity, and duration), and the outcomes of interest (e.g., blood pressure, lipid profiles, glucose levels, and insulin sensitivity). Discrepancies between reviewers were resolved through discussion and if consensus was not reached a third reviewer (Author 3) was consulted to make a final decision.

### 2.5. Quality Assessment

The methodological quality of the included studies was assessed using the Physiotherapy Evidence Database (PEDro) scale, which scores studies from 0 to 10 based on their methodological rigor. Studies scoring 9–10 were considered of “excellent” quality, scores between 6–8 were rated as “good”, and studies scoring 4–5 were deemed “fair”. Studies scoring below 4 were excluded from the review due to methodological concerns. While the inclusion of studies with PEDro scores of 4–5 may limit the strength of the evidence, such studies were included because they contributed valuable information to the research question. However, these studies’ findings were interpreted with caution due to their lower methodological quality [19].

### 2.6. The Evidence Levels

The strength of the evidence was classified according to the Sackett scale [20].

Level 1a (strong evidence): At least two high-quality RCTs with similar results.

Level 1b (moderate evidence): One high-quality RCT.

Level 2a (limited evidence): At least one fair-quality RCT.

Level 2b (limited evidence): At least one poor-quality RCT.

### 2.7. PRISMA Flowchart

A PRISMA flowchart was used to visually represent the study selection process, showing the number of studies identified, screened, assessed for eligibility, and ultimately included in the review. Reasons for exclusions were recorded at each stage of the process, including irrelevant study designs, failure to meet inclusion criteria, or insufficient reporting of outcomes.

### 2.8. Data Analysis and Synthesis

Due to the variations in exercise protocols across the studies included in this review, some degree of variability (heterogeneity) in the results was anticipated, which may affect the overall interpretation. Although a meta-analysis was not possible because of considerable differences in both the exercise modalities and participant characteristics, we performed a descriptive assessment to examine the heterogeneity. A qualitative synthesis was used to summarize the findings, grouping studies by the type of exercise intervention (e.g., aerobic exercise, resistance training, HIIT, MICT, or CT) and the cardiovascular risk factors measured (e.g., lipid profiles, blood pressure, glucose, and insulin sensitivity).

In addition, we examined potential sources of variability, including factors such as exercise intensity, duration, frequency, and characteristics of the study participants (e.g., age, BMI, and baseline health status). For instance, variations in exercise intensity, such as the differences between HIIT and MICT, could influence cardiovascular risk factors differently, and we incorporated these aspects into the synthesis of the results.

## 3. Results

### 3.1. Study Selection

**Figure 1:**
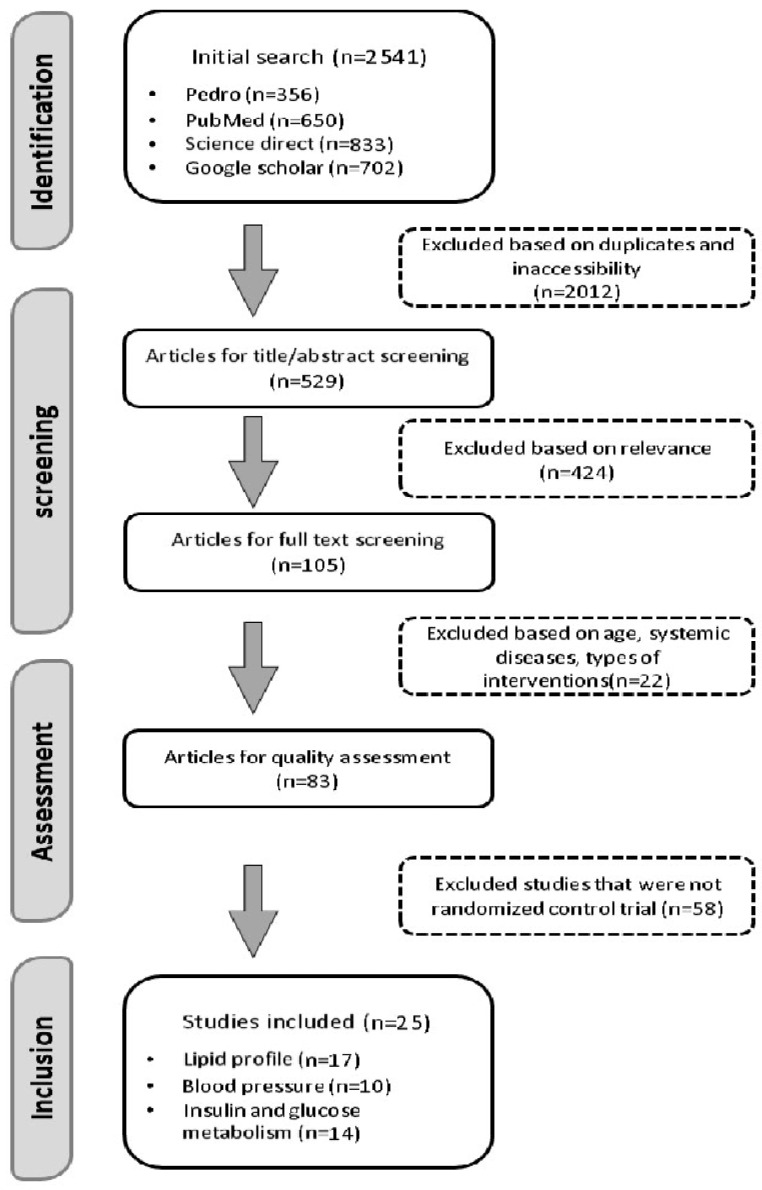
PRISMA flow chart of search strategy. This figure illustrates the selection process of the reviewed studies.

A total of 2541 studies were initially identified. After removing duplicates and irrelevant studies, 529 studies remained. Following screening of titles and abstracts, 105 studies were selected for full-text review. Of these, 22 were excluded for failing to meet the inclusion criteria, leaving 83 studies. After further excluding non-RCT studies, 25 articles were ultimately included in the final review. The study selection process is depicted in Figure 1.

### 3.2. Characteristics of the Included Studies

The 25 studies included 2374 male participants aged 18 to 72 years, all classified as overweight or obese (BMI ≥ 25). None of the participants had a history of cardiovascular disease or other chronic conditions. BMI classifications were used to define overweight (25.0 to <30 kg/m^2^) and obese (≥30 kg/m^2^) participants. The details of the included studies’ quality are presented in Table 1.

Due to significant heterogeneity across the studies in terms of exercise protocols (type, frequency, duration, and intensity), it was not feasible to conduct a meta-analysis. A qualitative synthesis was therefore performed as presented in Table 2.

### 3.3. Characteristics of Exercise Programs

Endurance Training (ET): Training regimens varied between six [43] and 22 [38] weeks, performed three to five days per week at moderate intensity.

HIIT: The training duration ranged from 3 [37] weeks through to 6 [24], 8 [22,44], 12 [26], and 16 [35] weeks, with one to three sessions per week at high intensity (80–100%). Session durations ranged from 30 to 60 min.

RT: Interventions varied from eight [27,33,36] to 12 [14,34] and 24 [41] weeks at moderate to high intensity, performed three times per week, with each session lasting approximately 60 min.

CT: Training programs lasted between 12 [23,25,29,32,42], 22 [38], and 24 [28,30] weeks, performed three days per week at moderate intensity, and each session lasted between 60 to 120 min.

Strength Training: Strength training was examined in one study with 22 [38] week protocols, performed three times per week at 90% of one-repetition maximum (1RM).

### 3.4. Outcomes

#### 3.4.1. Blood Pressure

Studies and Participants: A total of 818 participants from 10 studies, aged 18–50+, with overweight or obesity, were analyzed.

Exercise Interventions: Interventions included HIIT, MICT (3 studies) [22,24,26], CT (3 studies) [23,25,29], RT (1 study) [14], aerobic exercise (2 studies) [39,43], boxing (1 study) [21], and tai chi (1 study) [29].

Findings: HIIT consistently demonstrated significant reductions in blood pressure (BP) across all studies. MICT showed no significant difference compared to HIIT. One RT study and interventions like boxing and tai chi also reported significant reductions. Aerobic exercise demonstrated non-significant reductions in BP.

Quality and Evidence: HIIT and MICT provided limited evidence (Level 2a) for BP reduction based on three fair-quality RCTs. CT showed moderate evidence (Level 1b), supported by one high-quality and two fair-quality RCTs. Aerobic exercise showed limited evidence (Level 2a) from two fair-quality RCTs (Table 3).

#### 3.4.2. Blood Insulin and Glucose

Studies and Participants: Fourteen RCT studies with 456 total participants were reviewed.

Exercise Interventions: HIIT, MICT [22,35,37,44], CT [28,30,32,42], RT [14,27,33,34,36], and aerobic exercise [31] were analyzed.

Findings: HIIT led to significant reductions in insulin and glucose in only one of five studies. RT demonstrated significant improvements in all five studies, while aerobic exercise showed no significant changes. CT produced reductions in insulin and glucose in two out of three studies.

Quality and Evidence: HIIT and MICT demonstrated limited evidence (Level 1b) for reducing insulin and glucose. RT provided moderate evidence (Level 2a) from five fair-quality RCTs. CT also showed moderate evidence (Level 2a) based on three fair-quality RCTs (Table 4 and Table 5).

#### 3.4.3. Lipid Profile

Studies and Participants: Seventeen RCT studies involving 1100 participants with obesity or overweight.

Exercise Interventions: HIIT, MICT [22,24,35,37], CT [23,25,28,29,42], RT [14,33,41], aerobic exercise [31,39,40,43], strength training [38], and tai chi [29] were included.

Finding:

HDL: HIIT showed significant increases in HDL in one of five studies, while MICT and RT yielded mixed results. CT showed improvements in HDL in two of five studies, and aerobic exercise showed positive effects in three of five studies.

LDL: HIIT showed significant reductions in LDL in one of three studies, with similar results in MICT. RT showed mixed results, while CT showed reductions in LDL in two of four studies.

Triglycerides (TG): HIIT and MICT did not significantly reduce TG. However, RT and strength training demonstrated positive outcomes.

Total Cholesterol (TC): HIIT and RT did not show significant reductions in TC, while CT and aerobic exercise resulted in significant reductions.

Quality and Evidence:

HDL: HIIT and MICT demonstrated moderate evidence (Level 1b) for improvements in HDL.

LDL and TG: RT and CT showed mixed evidence; however, CT provided moderate evidence (Level 1b) based on high-quality RCTs.

TC: Aerobic exercise and CT demonstrated moderate evidence (Level 1b) for TC reduction.

## 4. Discussion

This systematic review aimed to assess the effects of various exercise modalities—HIIT, MICT, RT, CT, and aerobic exercise—on cardiovascular risk factors in overweight and obese males (Table 6, Table 7 and Table 8). The overall results indicate that exercise interventions generally resulted in positive changes in BP, glucose levels, insulin sensitivity, and lipid profiles. However, significant heterogeneity across studies limits the ability to draw definitive conclusions. This variability likely arises from differences in exercise protocols (e.g., duration, intensity, and frequency), study designs, and participant characteristics.

Inter-study variability constitutes a key factor in interpreting these findings. Divergences in exercise modalities, encompassing duration, intensity, frequency, and specific type (e.g., HIIT, MICT, or RT), emerged as significant sources of this heterogeneity. For instance, HIIT interventions typically spanned durations of 6 to 16 weeks, with 1 to 3 sessions per week. In contrast, MICT programs were generally longer, extending from 12 to 24 weeks, and often involved 3 to 5 sessions per week. Furthermore, the intensity of HIIT was substantially greater than that of MICT or aerobic exercise, which may explain the more pronounced improvements in BP and insulin sensitivity observed in some HIIT studies. While MICT demonstrated beneficial effects, these were generally less pronounced compared to the outcomes observed with HIIT.

Most of the included studies were assessed as having fair quality. Some studies presented with limitations such as small sample sizes, short follow-up periods, or methodological shortcomings (e.g., inadequate randomization or blinding). These methodological limitations may have influenced the observed findings and potentially reduced the generalizability of the results. Notably, studies investigating HIIT demonstrated consistent benefits in reducing BP, whereas those examining RT and aerobic exercise exhibited more varied results. This variability in study quality raises concerns regarding the robustness of the evidence, particularly for RT and aerobic exercise, where smaller sample sizes and methodological flaws may have exerted a significant impact on the observed outcomes.

Several studies reported non-significant findings, particularly with respect to glucose and lipid profiles. This observation suggests that exercise interventions alone, in the absence of concomitant dietary or pharmacological interventions, may not be sufficient to induce significant improvements in these crucial cardiovascular risk factors within the context of obesity. However, it is important to emphasize that non-significant results do not necessarily imply the ineffectiveness of the exercise interventions. The lack of statistically significant effects could be attributed to factors such as small sample sizes or short intervention durations, which may not have been sufficient to allow for the observation of meaningful clinical changes. Furthermore, the modest improvements observed in some studies may reflect the inherent complexities associated with managing metabolic conditions in individuals with obesity.

Participant demographics, including age, BMI, and baseline health status, also exerted a significant influence on the observed outcomes. For instance, studies involving older adults or individuals with more severe obesity demonstrated distinct responses to exercise interventions compared to younger individuals with milder obesity. This observed demographic variability underscores the critical need for targeted research that meticulously accounts for individual differences in exercise response.

### 4.1. Blood Pressure

High-intensity interval training was associated with a consistent reduction in BP in males with obesity, with similar effects observed for MICT. However, HIIT appeared to produce somewhat greater improvements, particularly in systolic BP, compared to other exercise modalities, which is consistent with findings from previous systematic reviews and original studies [16,45]. These improvements are likely attributable to a reduction in peripheral vascular resistance, a consequence of enhanced endothelial function and increased vascular shear stress induced by high-intensity exercise [21]. It is noteworthy that, despite the considerably shorter duration of HIIT interventions compared to other exercise modalities (for example, one study implemented five 1 min bouts with 1 minute of active recovery), reductions in systolic blood pressure were still observed. Furthermore, variations in exercise frequency, ranging from one to three days per week, did not significantly alter the observed outcomes (reviewed in a single study).

Resistance training (RT) demonstrated a more limited effect on blood pressure (BP) compared to other interventions. The modest improvements observed in RT studies may be attributed to several factors, including the small number of studies (*n* = 1) and its relatively brief duration (≤12 weeks). While shorter RT programs may not lead to significant BP reductions, previous research indicates that longer RT interventions might yield greater improvements in BP [46]. The variability in RT protocols, including intensity, volume, and short durations, likely limited BP improvements. Low-intensity RT may also be insufficient for significant cardiovascular benefits, highlighting the importance of considering program duration and intensity for BP management in obesity.

Combined training (CT) may offer some benefits for BP, although the underlying mechanisms are not fully understood. The possible reasons for the observed BP improvements include changes in arterial stiffness or reductions in plasma epinephrine levels. The variability in CT protocols, such as different combinations of aerobic and resistance training modalities complicates the ability to draw definitive conclusions regarding its effectiveness for BP management. Some combined exercise protocols were implemented concurrently, while others were conducted in separate sessions. Additionally, the duration of each session varied, ranging from 60 min to 120 min. In comparison, aerobic exercise alone typically shows more limited effects on BP, with some studies indicating no significant reductions. This finding is consistent with other research, suggesting that the impact of aerobic exercise on BP may vary, potentially influenced by factors such as the intensity and duration of exercise, or the integration of different training modalities [47,48].

It is crucial to acknowledge that the quality of the included studies exhibited variability. The studies focusing on HIIT had a higher quality rating (1b) compared to the other studies (2a). Furthermore, two studies investigating combined training and endurance training had small sample sizes, with only 6 and 9 participants in each group, respectively. These small sample sizes may have influenced the findings and affected the overall reliability of the results.

### 4.2. Blood Insulin and Glucose

HIIT was linked to slight improvements in insulin resistance in obese men, though its impact on glucose and insulin regulation was not significant. These findings may be influenced by variations in participant characteristics, such as age. The participants in these studies ranged in age from 18 to 50 years. It is likely that older men might respond differently to exercise compared to younger individuals, which could explain the differences in the study outcomes. Some studies reported no significant effects on glucose metabolism, suggesting that other factors, such as alterations in body composition, may exert a more substantial influence on improving insulin sensitivity than exercise intensity alone [49]. Although some studies have noted a reduction in body fat percentage or BMI, others have found no significant changes in body fat levels, as several systematic reviews have concluded that exercise interventions alone may not induce clinically significant weight loss [50]. Resistance training (RT) demonstrated beneficial effects on insulin sensitivity and blood insulin levels, while exhibiting minimal effects on glucose tolerance. These improvements may be attributed to an increase in lean body mass and enhanced muscle metabolic properties [51]. The effectiveness of RT in improving insulin levels and sensitivity appears to be influenced by the intensity and volume of the exercises. Non-periodized protocols (characterized by steady intensity and volume) and linear periodization (which involves a gradual increase in intensity and a decrease in volume) did not significantly affect insulin levels. In contrast, an undulating periodized protocol, which incorporates a weekly progression in exercise intensity, resulted in significant improvements in insulin regulation (reviewed in a single study). Combined training (CT), incorporating both aerobic and resistance exercises, also exerted a positive influence on insulin and glucose regulation, whereas aerobic exercise alone exhibited limited effects on fasting glucose [48]. This suggests that a CT approach may offer more comprehensive benefits for glucose regulation compared to any single exercise modality. It is important to note that CT protocols reviewed in this study had a longer duration compared to aerobic exercise alone (24 weeks vs. 8 weeks). Additionally, the duration of each training session in the CT studies was longer than that in aerobic exercise (60 min vs. 30 min). These differences in both training duration and session length may influence the results, independent of the type of exercise performed.

Resistance training (RT) and CT studies included in this review exhibited higher methodological quality than the HIIT and IT studies (1b VS. 2a), it is important to note that the RT studies involved younger participants (ranging from 23 to 36 years) and showed less age variability. In contrast, the studies with HIIT and ET had a wider age range (18 to 68 years), which may have contributed to greater variability in the effects of exercise.

### 4.3. Lipid Profile

This review found that HIIT exhibited minimal effects on most lipid factors, including triglycerides and total cholesterol, while demonstrating slight improvements in HDL and LDL levels in males with obesity. These findings are consistent with the observations of Batacan et al. (2017), who suggested that longer-duration HIIT interventions may be required to observe significant alterations in lipid profiles [52]. The majority of studies examining the effect of HIIT on lipid profiles were short-term and utilized very high-intensity protocols, such as 10 repetitions of 1 min running bouts at 80–90% HR max. The lack of substantial lipid improvements may be attributed to HIIT’s potential to reduce fatty acid release into the bloodstream by limiting blood flow to adipose tissue, although further research is necessary to elucidate this mechanism [53]. Furthermore, the variability in HIIT protocols, including differences in intensity, duration, and frequency across studies, likely contributes to the mixed results observed (e.g., 10 one-minute bouts vs. 5 two-minute bouts, each performed at varying intensities). For example, the short-term nature of many HIIT interventions, as well as the use of high-intensity protocols, may limit the long-term effects on lipid metabolism.

Resistance training (RT) exhibited minimal effects on lipid profiles, with no discernible changes in total cholesterol. Previous research on RT has demonstrated mixed results, likely attributable to variations in genetic factors, age, and intervention duration [54]. The RT studies included in this review involved participants aged between 20 and 72 years, with intervention durations ranging from 8 to 24 weeks. This variation in both age and the length of the interventions across the studies makes it difficult to draw clear conclusions about the effects of RT on lipid profiles. In addition, another key observation in the studies is that the intensity of the exercises appears to be more influential than the duration. For instance, 24 weeks of RT at an intensity ranging from 50% to 80% of 1 repetition maximum did not significantly impact the lipid profile. In contrast, 12 weeks of higher-intensity RT (65–85% of 1 repetition maximum) resulted in a more pronounced improvement in lipid profiles. The inconsistencies in these factors may contribute to variability in the outcomes, highlighting the importance of more consistent study designs to better understand the potential influence of RT on lipid metabolism.

Combined training (CT) programs seem to have a moderate, indirect effect on lipid profiles, particularly enhancing HDL cholesterol levels. These outcomes are attributed to both the direct impact of these interventions on lipid metabolism observed in three studies and their contribution to reducing body fat percentage, which may also play a role in improving lipid profiles. In these three studies, both resistance and aerobic exercises were performed simultaneously within a single session, with the intensity of the resistance exercises being notably high. Studies by Ho et al. [55] and Park et al. [25] reported significant reductions in body weight, BMI, and body fat percentage in individuals engaging in CT programs. This combined approach is particularly relevant for reducing abdominal fat, a critical factor due to its strong association with an increased risk of type 2 diabetes and cardiovascular disease. One potential mechanism underlying these beneficial effects may involve improvements in the leptin–catecholamine axis and reductions in plasma epinephrine levels, contributing to enhanced body composition and improved cardiovascular function [56]. It is important to note that the findings regarding the impact of this intervention on the lipid profile are based on evidence of relatively high quality (1a), allowing for a more reliable interpretation of the results.

Aerobic exercise has been shown to be associated with modest improvements in lipid factors, including a potential increase in HDL cholesterol levels, consistent with findings from several studies demonstrating improvements in lipid profiles, specifically an increase in HDL cholesterol [57]. Given the role of aerobic exercise in reducing body fat percentage, as reported in three studies, it may contribute to improvements in lipid profiles through this mechanism. Based on the studies reviewed in this paper, it appears that three sessions of aerobic exercise per week may not be sufficient to induce significant changes in lipid profile. In contrast, protocols with higher frequency (4 to 5 days per week) and progressive elements seem to be more effective. Additionally, it seems that a minimum session duration of 45 min may be necessary to achieve optimal effects on lipid metabolism. In this context, factors such as exercise frequency, progression, and session duration may play a more influential role in improving lipid profiles than the intensity or overall duration of the exercise protocol. However, it is important to acknowledge that the participants in these studies were primarily over 45 years of age, with considerable variation in their ages. This factor may influence the results, particularly when compared to younger populations. Furthermore, the overall quality of the included studies was rated at 2a level, which provides a moderate degree of confidence in the evidence. As a result, the findings should be viewed with caution, taking into account the possibility of biases and confounding variables.

### 4.4. Limitations

This study has several limitations. Firstly, the inclusion criteria were limited to English-language, peer-reviewed journals. Secondly, the literature search was restricted to four databases (PubMed, PEDro, ScienceDirect, and Google Scholar), which may have resulted in the exclusion of relevant studies. Incorporating additional databases (e.g., EMBASE and Scopus) and conducting comprehensive manual searches could enhance the comprehensiveness of the review. Thirdly, some cardiovascular risk factors, such as fibrinogen, were excluded from the analysis due to a limited number of studies investigating their relationship with exercise in this population. Fourthly, several studies did not report their results with sufficient clarity, and attempts to contact the corresponding authors for clarification were unsuccessful.

Future research also should investigate the effects of various exercise protocols on a broader range of cardiovascular risk factors, including fibrinogen and blood viscosity, in individuals with obesity. Studies should also consider more precise evaluations of key variables, examine different obesity classifications (e.g., based on body fat percentage), and focus on more homogeneous populations to minimize confounding factors.

## 5. Practical Recommendation

**Figure 2:**
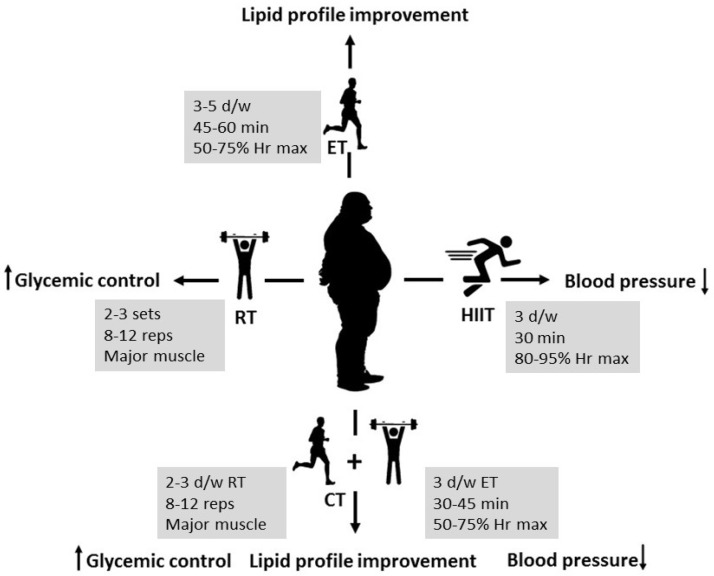
Summary of the results (HIIT: high-intensity interval training; RT: resistance training; CT: combined training; ET: endurance training; W: week; d/w: days per week; Hr: heart rate, reps: repetitions).

This review highlights the effectiveness of various exercise modalities in reducing cardiovascular risk factors in obese males. Results of this systematic review indicate that HIIT effectively reduces systolic blood pressure, but may not be suitable for individuals with cardiovascular issues. In addition, resistance training improves glycemic control and insulin sensitivity, especially with longer interventions, while aerobic exercise is essential for improving HDL cholesterol and body composition. Applying exercise programs based on evidence in clinical settings, incorporating structured exercise plans into workplace and community wellness programs, and customizing these plans for specific groups, such as older adults or individuals with severe obesity, can improve health outcomes and increase participation in physical activity. Policymakers should encourage physical activity programs and work with insurance companies to increase participation. Future research should focus on how to maintain long-term participation, combine interventions, assess cost-effectiveness, and include a wider range of populations to make the findings more applicable (Figure 2).

## 6. Conclusions

This systematic review indicates that exercise training can positively influence cardiovascular health in males with obesity, though the effects depend on the exercise type and the specific cardiovascular risk factors being addressed. High-intensity interval training (HIIT) has shown some effectiveness in reducing blood pressure, with certain studies reporting up to a 10 mmHg reduction in systolic blood pressure after 12 weeks. However, the evidence supporting HIIT as the most effective approach for blood pressure reduction is not consistent across all studies. Aerobic exercise may require longer or more intense programs to produce substantial improvements in lipid profiles, with some studies showing modest increases, particularly in HDL cholesterol levels. Resistance training (RT) has shown potential in improving glycemic control, though the findings are inconclusive. Factors such as age, gender, race, baseline cardiovascular risk, and the specifics of the RT regimen may influence the effectiveness of RT in improving glucose metabolism and these aspects require further investigation. Combined training (CT), which includes both aerobic and resistance exercises, may have some potential for improving cardiovascular risk factors, such as lipid profiles, glucose levels, and insulin sensitivity. However, the evidence remains mixed; for example, only two out of five studies on CT reported significant improvements in HDL levels. Despite these inconsistencies, combined training remains a practical approach, with consistent participation (approximately 60 min, three times per week) potentially yielding cardiovascular health benefits. Future research should focus on more consistent age ranges, have higher-quality designs, and include both sexes to better understand the effects across genders. Additionally, long-term studies exploring the impact of various exercise modalities, considering baseline fitness and pre-existing health conditions, are needed.

## Figures and Tables

**Table 1:** The quality of the reviewed studies using the PEDro scale.

Author	#1 *	#2	#3	#4	#5	#6	#7	#8	#9	#10	#11	Total	Quality
Cheema [21]	1	1	1	1	0	0	1	0	1	1	1	7/10	Good
Chin [22]	0	1	0	1	0	0	0	0	0	1	1	4/10	Fair
Croymans [14]	0	1	0	1	0	0	0	0	0	1	1	4/10	Fair
Gahete [23]	1	1	0	1	0	0	1	0	0	1	1	5/10	Fair
Neto [24]	1	1	0	1	0	0	0	0	0	1	1	4/10	Fair
Park [25]	0	1	0	1	0	0	0	0	0	1	1	4/10	Fair
Reljic [26]	1	1	0	1	0	0	1	0	0	1	1	6/10	Good
Ahmadizad [27]	0	1	0	1	0	0	0	0	0	1	1	4/10	Fair
Bonfante [28]	0	1	0	1	0	0	0	0	0	1	1	4/10	Fair
Siu [29]	0	1	0	1	0	0	0	0	0	1	1	4/10	Fair
Duft [30]	0	1	0	1	0	0	0	0	0	1	1	4/10	Fair
Kim [31]	0	1	0	1	0	0	0	1	0	1	1	5/10	Fair
Kim [32]	0	1	0	1	0	0	0	1	0	1	1	5/10	Fair
Kolahdouzi [33]	0	1	0	1	0	0	0	0	0	1	1	4/10	Fair
Nikseresht [34]	0	1	0	1	0	0	0	0	0	1	1	4/10	Fair
Poon [35]	1	1	0	1	0	0	0	0	0	1	1	4/10	Fair
Safarzade [36]	1	1	0	1	0	0	0	0	0	1	1	4/10	Fair
Smith-Ryan [37]	1	1	0	0	0	0	0	0	0	1	1	3/10	poor
Moraleda [38]	1	1	0	1	0	0	0	0	0	1	1	4/10	Fair
Saremi [39]	1	1	0	1	0	0	0	0	0	1	1	4/10	Fair
Varady [40]	0	1	0	1	0	0	0	0	0	1	1	4/10	Fair
Vincent [41]	0	1	0	1	0	0	0	1	0	1	1	5/10	Fair
Saeidi [42]	1	1	0	1	0	0	0	1	1	1	1	6/10	Good
Touli [43]	1	1	0	1	0	0	0	0	1	1	1	5/10	Fair
Rezaeimanesh [44]	1	1	0	1	0	0	0	0	1	1	1	5/10	Fair

* The first item does not contribute to total score.

**Table 2:** Sackett’s levels of evidence for outcomes.

Outcomes	Exercise Modality	Study	Level of Evidence
Blood pressure	HIIT RT CT ET	3 RCTs 1 RCTs 3 RCTs 2 RCTs	1b 2a 2a 2a
Glucose and insulin	HIIT RT CT ET	3 RCTs 5 RCTs 3 RCTs 2 RCTs	2a 1b 1b 2a
Lipid profile	HIIT RT CT ET	3 RCTs 3 RCTs 5 RCTs 3 RCTs	2a 2a 1a 2a

**Table 3:** Summary of study characteristics and findings on the effects of exercise training on blood pressure in overweight or obese males.

Authors	Participants	Intervention	Comparison	Outcome (Measure)	Results
Hematinezhad Touli, [43]	AE: *n* = 10 CON: *n* = 10 Age: 28–35 BMI: 25–30	6 w 3 d/w 45 min 50–70% HR max	no exercise	BP (digital wrist monitor)	BMI (+) ↓ Body fat (+) ↓ SBP (+) ↓ DBP (+) ↓
Cheema, [21]	Bx: *n* = 6 CON: *n* = 6 Age > 18 BMI = 32	12 w 4 d/w 50 min/d	no exercise	BP (manually at the brachial and radial artery)	BMI = 30.5 (−) SBP (+) ↓ DBP (+) ↓
Chin, [22]	MICT: *n* = 9 HIIT 3: *n* = 14 HIIT 2: *n* = 10 HIIT 1: *n* = 9 CON: *n* = 14 Age: 22.8 ± 3.1 BMI: 26.4 ± 2.9	8 W 1–3 d/w 30 min/d MICT 3 d/w HIIT 3 d/w HIIT 2 d/w HIIT 1 d/w	no exercise	BP (electronic sphygmomanometer)	Time × group interaction: SBP (+) ↓ DBP (+) ↓ BMI (+) ↓ Body weight (+) ↓ All exercise group: SBP (+) ↓
Croymans, [14]	RT: *n* = 28 CON: *n* = 8 Age: 20–23 BMI: 31.4	12 w 3 d/w 1 h/day	light physical activities without RT	BP (SphygmoCor system)	Body fat (+) ↓ SBP (+) ↓ DBP (+) ↓
Gahete, [23]	CT: *n* = 6 CON: *n* = 6 Age: 42.5 BMI > 30	12 w 3 d/w 60 min/d Concurrent training	no exercise	BP (automatic monitor)	Weight (+) ↓ BMI (+) ↓ BP (+) ↓
Neto, [24]	HIIT: *n* = 13 MICT: *n* = 13 CON: *n* = 6 Age: 18–35 BMI ≥ 30	6 w 3 d/w 30–60 min HIIT: 10 efforts at 100% of MAV/1-min passive recovery MICT: 30 min to 65% of the MAV	no exercise	BP (aneroid sphygmomanometer)	In both HIIT and MICT: BMI (−) Visceral fat (−) SBP (+) ↓ DBP (−)
Park, [25]	CT: *n* = 10 CON: *n* = 10 Age: 68.8 BMI of >25	12 W 3 d/w 90–120 min Combined training	no exercise	BP (automatic sphygmomanometer)	BP (+) ↓ Body weight (−) BMI (−)
Reljic, [26]	HIIT: *n* = 30 CON: *n* = 19 Age ≥ 18 BMI ≥ 30	12 w 2 d/w HIT: 5 efforts of 1 min at 80–95% HRmax/1 min of low-intensity recovery	no exercise	BP (automatic upper-arm monitor)	Body weight (−) BMI (−) SBP (+) ↓ DBP (+) ↓
Siu, [29]	CT: *n* = 181 Tai chi: *n* = 181 CON: *n* = 181 Age ≥ 50	12 W CT: aerobic exercise and strength training or Tai chi	no exercise	BP (NA)	Both CT and Tai chi: Body weight (+) ↓ BP (−)
Saremi, [39]	ET: *n* = 9 CON: *n* = 9 Age: 43.1 + 4.7 BMI > 25	12 w 5 d/w 50–60 min ET: progressive aerobic training	usual lifestyle	BP (mercury sphygmomanometer)	Body weight (+) ↓ BMI (+) ↓ SBP (+) ↓ DBP (−)

HIIT: high-intensity interval training; MICT: moderate-intensity continuous training; RT: resistance training; ET: endurance training; CT: combined training; CON: control group; BP: blood pressure; BX: boxing; SBP: systolic blood pressure; DBP: diastolic blood pressure; G: glucose; IN: insulin; IR: insulin resistance; (+): significantly different from control; (−): no differences between intervention and control groups; ↑: increase; ↓: reduction.

**Table 4:** Summary of study characteristics and findings on the effects of exercise training on blood insulin and glucose in males with overweight or obesity.

Authors	Participants	Intervention	Comparison	Outcome (Measure)	Results
Saeidi, [42]	CT: *n* = 17 CON: *n* = 17 Age: 27.6 ± 8.4 BMI: > 30	12 w 3 d/w 60 min concurrent strength and endurance training	no exercise	IR (HOMA-IR) IN (ELISA kit) G (colorimetric enzymatic kit)	Body weight (+) BMI (+) ↓ IN (−) G (−) IR (−)
Rezaeimanesh, [44]	HIT: *n* = 12 CON: *n* = 12 Age: 19–25 BMI: > 25	8 w 3 d/w HIT: 1 min at 85–88% of HR max followed 2 min at 50–55% HR max (10 times)	no exercise	G (Glucose kit) IR (HOMA-IR) Insulin (ELISA kit)	Within group: Body weight (+) BMI (+) ↓ Between group: Body weight (−) BMI (−) G (−) IR (+) ↓ IN (−)
Ahmadizad, [27]	NP: *n* = 8 LP: *n* = 8 DUP: *n* = 8 CON: *n* = 8 Age: 23.4 ± 0.6 BMI = 25–29.9	8 W 3 d/w nonperiodized group: steady resistance exercise intensity and training volume linear periodized group: gradually increasing exercise intensity and decreasing training volume daily undulating periodized group: fluctuating exercise intensity and training volume from day to day within a week.	no exercise	Plasma IN (ELISA kit) Plasma G (enzymatic colorimetric method) IR (HOMA-IR)	Both NP and LP: Body fat (+) ↓ BMI (−) IN (−) G (−) IR (−) DUP: In (+) ↓ G (−) IR (−)
Bonfante, [28]	CT: *n* = 12 CON: *n* = 10 Age: 49.13 ± 5.75 BMI: 30.86 ± 1.63	24 w 3 d/w CT: Strength training (3 sets of 6–10 maximal rep) and endurance training (walking/ running at 55–85% of VO2peak)	no exercise	G levels (automatic anal 2008yser and a commercially available kit) IN (chemiluminescence) IR and IS (HOMAbeta/HOMA-IR)	BMI (−) Body weight (−) HDL (+) ↑ LDL (+) ↓ IN (+) ↓ G (+) ↓ TC (+) ↓ IR (+) ↓ IS (+) ↑ G (+) ↓
Chin, [22]	MICT: *n* = 9 HIIT 3: *n* = 14 HIIT 2: *n* = 10 HIIT 1: *n* = 9 CON: *n* = 14 Age: 22.8 ± 3.1 BMI: 26.4 ± 2.9	8 W 1–3 d/w 30 min/d MICT 3d/w HIIT 3d/w HIIT 2d/w HIIT 1d/w	no exercise	Fasting IN (enzyme-linked immunosorbent assay) Fasting G (commercial test kit)	Time × group interaction: Fasting G (−) Fasting IN (−) IR (−)
Croymans, [14]	RT: *n* = 28 CON: *n* = 8 Age: 20–23 BMI: 31.4	12 w 3 d/w 60 min	light physical activities without RT	Fasting IN (enzyme-linked immunosorbent assay)	Body fat (+) ↓ IN (+) ↓
Duft, [30]	CT: *n* = 11 CON: *n* = 11 Age: 48.2 BMI: 31	24 w 3 d/wk 60 min Combined training: 30 min resistance 30 min aerobic	no exercise	Serum G (automatic chemistry analyzer), Serum IN (electrochemiluminescence)	Fat mass (+) ↓ Serum G (+) ↓ Serum IN (−)
Kim, [31]	ATG: *n* = 29 CON: *n* = 10 Age: 26.6 BMI: 28.19	8 w 4 d/w ATG: a treadmill running at 65–75% VO2max to burn approximately 600 Kcal per session	no exercise	Fasting G (ADVIA 1650 Chemistry system) Fasting IN (electro chemiluminescence immune assay) IR (HOMA-IR)	BMI (+) ↓ Body weight (+) ↓ Fasting G (−) Fasting IN (−) IR (+) ↓
Kim, [32]	CT: *n* = 10 CON: *n* = 10 Age: 68.8 BMI > 25	12 w 3 d/w CT: Three kinds of combined exercise interventions for 90–120 min: elastic resistance exercise, aerobic exercise on a treadmill, and aerobic exercise on a bicycle)	no exercise	Fasting G (enzymatic kinetic assay) Fasting IN (electro chemiluminescence immunoassay) IR (HOMA-IR)	Fat mass (+) ↓ Body weight (+) ↓ IN (+) ↓ IR (+) ↓ G (−)
Kolahdouzi, [33]	RT: *n* = 13 CON: *n* = 13 Age: 23 ± 3.2 BMI: 30.67 ± 3.06	8 w 3 d/w 60 min RT: 65–85% of 1 repetition maximum	no exercise	Fasting IN (ELISA) Fasting G (Glucose oxidize) IR (HOMA-IR)	BMI (+) Body weight (+) IN (+) − IR (+) − G (−)
Nikseresht, [34]	RT: *n* = 12 AIT: *n* = 12 CON: *n* = 10 Age: 34–46 years BMI >25	12 weeks 3 d/w RT: 40–65 min of weight training at different intensities with flexible periodization AIT: 4 sets of 4 min running at 80–90% of HR max, with 3-min recovery	no exercise	Serum G (glucose oxidase method) serum IN (ELISA) IR (HOMA-IR)	Both training group: Fat mass (+) ↓ G (−) IN (+) ↓ IR (+) ↓
Poon, [35]	HIIT: *n* = 11 MICT: *n* = 10 HIIT-MICT: *n* = 11 CON: *n* = 10 age: 42 ± 5 BMI: 26.3 ± 2.1	16 w 3 d/w HIIT: 12 × 1-min running bouts at 80–90% HR max with 1-min active recovery at 50% HR max MICT: 40-min brisk walk at 65–70% HR max HIIT-MICT: alternating both method	no exercise	G (enzymatic amperometric method) IN (ELISA) IR (HOMA-IR)	BMI (+) ↓ Body weight (+) ↓ IN (−) IR (−) G (−)
Safarzade, [36]	RT: *n* = 14 CON: *n* = 14 age: 36 ± 7.7 BMI: 32.4 ± 4.5	8 w 3 d/w RT: 3 circuits in each session, with 12 stations per circuit and 8–10 repetitions per station	no exercise	G (enzymatic amperometric method) IR (HOMA-IR) IN (ELISA)	BMI (+) ↓ Body weight (+) ↓ IN (+) ↓ IR (+) ↓ G (−)
Smith-Ryan, [37]	HIIT 1: *n* = 10 HIIT 2: *n* = 10 CON: *n* = 5 Age: 18–50 BMI > 25	3 w 3 d/w HIIT 1: 10 × 1 min bouts at 90% power with 1 min rests HIIT 2: 5 × 2 min bouts with 1 min rests at undulating intensities (80–100%)	no exercise	fasting G and IN (enzymatic assays for) IR (HOMA-IR)	Both group: Body fat (−) IN (+) ↓ IR (+) ↓ G (−)

HIIT: high-intensity interval training; MICT: moderate-intensity continuous training; RT: resistance training; ET: endurance training; CT: combined training; CON: control group; BP: blood pressure; SBP: systolic blood pressure; DBP: diastolic blood pressure; G: glucose; IN: insulin; IR: insulin resistance; (+): significantly different from control; (−): no differences between intervention and control groups; ↑: increase; ↓: reduction.

**Table 5:** Summary of study characteristics and findings on the effects of exercise training on lipid profile in males with overweight or obesity.

Authors	Participants	Intervention	Comparison	Outcome (Measure)	Results
Saeidi, [42]	CT: *n* = 17 CON: *n* = 17 Age: 27.6 ± 8.4 BMI: > 30	12 w 3 d/w 60 min concurrent strength and endurance training	no exercise	TC and TG (enzymatic methods) HDL and LDL (photometric method)	Body weight (+) ↓ BMI (+) ↓ HDL (+) ↑ LDL (+) ↓ TC (+) ↓ TG (+) ↓
Hematinezhad Touli, [43]	AE: *n* = 10 CON: *n* = 10 Age: 28–35 BMI: 25–30	6 w 3 d/w 45 min 50–70% HR max	no exercise	Lipid profile (NA)	BMI (+) ↓ Body fat (+) ↓ TC (+) ↓ LDL (+) ↓ HDL (+) ↑
Bonfante, [28]	CT: *n* = 12 CON: *n* = 10 age: 49.13 ± 5.75 BMI: 30.86 ± 1.63	24 w 3 d/w CT: Strength training (3 sets of 6–10 maximal rep) and endurance training (walking/ running at 55–85% of VO2peak)	no exercise	TC, TG, and HDL (automatic anal2008yser and a commercially available kit) LDL (Friedewald).	BMI (−) Body weight (−) HDL (+) ↑ LDL (+) ↓ TC (+) ↓
Chin, [22]	MICT: *n* = 9 HIIT 3: *n* = 14 HIIT 2: *n* = 10 HIIT 1: *n* = 9 CON: *n* = 14 Age: 22.8 ± 3.1 BMI: 26.4 ± 2.9	8 W 1–3 d/w 30 min/d MICT 3 d/w HIIT 3 d/w HIIT 2 d/w HIIT 1 d/w	no exercise	TG and HDL (commercial test kit)	Time × group interaction: BMI (+) ↓ Body weight (+) ↓ Time × group interaction: HDL (−) TG (−)
Croymans, [14]	RT: *n* = 28 CON: *n* = 8 Age: 20–23 BMI: 31.4	12 w 3 d/w 60 min	light physical activities without RT	LDL (Friedewald equation) TC, HDL, and TG (the Olympus AU400 Chemistry Analyzer, Center Valley, PA, USA)	Body fat (+) ↓ TC (−) TG (−) LDL (−) HDL (−)
Gahete, [23]	CT: *n* = 6 CON: *n* = 6 Age: 42.5 BMI > 30	12 w 3 d/w 60 min/d Concurrent training	no exercise	TC, HDL (enzyme-linked immunesorbent assay)	Weight (+) ↓ BMI (+) ↓ HDL (−) TC (−)
Kim, [31]	ET: *n* = 29 CON: *n* = 10 Age: 26.6 BMI: 28.19	8 w 4 d/w ATG: a treadmill running at 65–75% VO2max to burn approximately 600 Kcal per session	no exercise	TG, TC, HDL, and LDL (ADVIA 1650 Chemistry system)	BMI (+) ↓ Body weight (+) ↓ HDL (+) ↑ LDL (+) ↓
Kolahdouzi, [33]	RT: *n* = 13 CON: *n* = 13 Age: 23 ± 3.2 BMI: 30.67 ± 3.06	8 w 3 d/w 60 min RT: 65–85% of 1 repetition maximum	no exercise	TC, HDL, LDL, and TG (enzymatically)	BMI (+) ↓ Body weight (+) ↓ HDL (+) ↑ LDL (+) ↓ TC (+) ↓ TG (+) ↓
Moraleda [38]	ST: *n* = 24 ET: *n* = 26 CT: *n* = 24 CON: *n* = 22 Age: 18–50 BMI >30 and <34.9	22 w 3 d/w ST: circuit involving the following eight exercises CT: a combination of cycle aerometry, treadmill or cross trainer work, plus weight training ET: involved the use of a treadmill, exercise bike or cross Training	habitual hospital clinical practice and diet	TC, LDL, HDL, and TG (enzymatic methods with Olympus reagents by automated spectrophotometry)	Body weight (+) ↓ HDL (−) LDL (−) TC (−) TG (−)
Neto, [24]	HIIT: *n* = 13 MICT: *n* = 13 CON: *n* = 6 Age: 18–35 BMI ≥ 30	6 w 3 d/w 30–60 min HIIT: 10 efforts at 100% of MAV/1-min passive recovery MICT: 30 min to 65% of the MAV	no exercise	TC, TG, and HDL (colorimetric)	In both HIIT and MICT: BMI (−) Visceral fat (−) HDL (−) TG (−) TC (−)
Park, [25]	CT: *n* = 10 CON: *n* = 10 Age: 68.8 BMI of >25	12 W 3 d/w 90–120 min Combined training	no exercise	TG, TC, HDL, and LDL (enzymatic colorimetric assay)	Body weight (−) BMI (−) Group x time interactions: TG (+) ↓ TC (+) ↓ LDL (+) ↓
Poon, [35]	HIIT: *n* = 11 MICT: *n* = 10 HIIT-MICT: *n* = 11 CON: *n* = 10 age: 42 ± 5 BMI: 26.3 ± 2.1	16 w 3 d/w HIIT: 12 × 1-min running bouts at 80–90% HR max with 1-min active recovery at 50% HR max MICT: 40-min brisk walk at 65–70% HR max HIIT-MICT: alternating both method	no exercise	TC (oxidase, esterase, and peroxidase colorimetric method) HDL (polyethylene glycol direct method) LDL (intra-assay CV) TG (enzymatic method)	BMI (+) ↓ Body weight (+) ↓ LDL (−) HDL (−) TC (−) IN (−)
Saremi, [39]	ET: *n* = 9 CON: *n* = 9 Age: 43.1 + 4.7 BMI > 25	12 w 5 d/w 50–60 min ET: progressive aerobic training	usual lifestyle	TC, TG, and HDL (enzymatic colorimetric) LDL (formula of Friedewald)	Body weight (+) ↓ BMI (+) ↓ TC (+) ↓ TG (+) ↓ LDL (+) ↓ HDL (−)
Siu, [29]	CT: *n* = 181 Tai chi: *n* = 181 CON: *n* =181 Age ≥ 50	12 W CT: aerobic exercise and strength training or Tai chi	no exercise	HDL (NA)	Both CT and Tai chi: Body weight (+) ↓ HDL (+) ↑
Smith-Ryan, [37]	HIIT 1: *n* = 10 HIIT 2: *n* = 10 CON: *n* = 5 Age: 18–50 BMI > 25	3 w 3 d/w HIIT 1: 10 × 1 min bouts at 90% power with 1 min rests HIIT 2: 5 × 2 min bouts with 1 min rests at undulating intensities (80–100%)	no exercise	TC, TG, and HDL (enzymatic assays for) VLDL and LDL (Friedwald’s equations)	Both group: Body fat (−) HDL (−) LDL (−) TC (−) TG (−)
Varady, [40]	ET: *n* = 15 CON: *n* = 15 Age: 35–65 BMI > 25	12 w 3 d/w ET: 45–60 min running at 60–75% of HR max.	no exercise	Plasma TC, LDL, HDL, and TG (enzymatic kits) LDL and HDL (linear polyacrylamide gel electrophoresis)	ET group: Body weight (+) HDL (+) ↑ LDL (−) TC (−) TG (−)
Vincent, [41]	RT: *n* = 10 CON: *n* = 10 Age: 60–72 BMI > 25	24 w 3 d/w RT: 8 to 13 repetitions at 50–80% of 1RM	no exercise	Plasma TC (commercial spectrophotometric method) HDL (commercial kit)	Fat mass (−) TC (−) HDL (−)

HIIT: high-intensity interval training; MICT: moderate-intensity continuous training; RT: resistance training; ET: endurance training; CT: combined training; CON: control group; BP: blood pressure; SBP: systolic blood pressure; DBP: diastolic blood pressure; G: glucose; IN: insulin; IR: insulin resistance; TC: total cholesterol, TG: triglyceride, LDL: low-density lipoprotein, HDL: high-density lipoprotein, (+): significantly different from control; (−): no differences between intervention and control groups; ↑: increase; ↓: reduction.

**Table 6:** Summary table (effects of exercise training on blood pressure in males with overweight or obesity).

Evidence Level	Key Findings	Number of Studies	Intervention
Limited (Level 2a)	Significant BP reductions	3	HIIT
Moderate (Level 1b)	Moderate BP reductions	3	CT
Limited (Level 2a)	Significant BP reductions	1	RT
Limited (Level 2a)	Non-significant BP reductions	2	ET

**Table 7:** Summary table (effects of exercise training on blood insulin and glucose in males with overweight or obesity).

Evidence Level	Key Findings	Number of Studies	Intervention
Limited (Level 1b)	Reductions in 1 study	5	HIIT
Moderate (Level 2a)	Reductions in 2 out of 3 studies	3	CT
Moderate (Level 2a)	Consistent improvements in all studies	5	RT
Moderate (Level 2a)	No significant changes observed	2	ET

**Table 8:** Summary table (effects of exercise training on lipid profile in males with overweight or obesity).

Evidence Level	LDL	HDL	Intervention
Moderate (Level 1b)	Reduction in 1/3 studies	Improvements in 1/5 studies	HIIT
Moderate (Level 1b)	Reduction in 2/4 studies	Improvements in 2/5 studies	CT
Limited (Level 2a)	Reduction in 1/2 studies	Improvements in 1/3 studies	RT
Moderate (Level 1b)	Not specified	Improvements in 3/5 studies	ET

## Data Availability

The data presented in this study are available within this manuscript.
